# Supplementary material for: Melanoma subpopulations that rapidly escape MAPK pathway inhibition incur DNA damage and rely on stress signalling
Source: Nat Commun. 2021 Mar 19;12:1747. doi: 10.1038/s41467-021-21549-x (PMC7979728; doi:10.1038/s41467-021-21549-x)
Supplement: Supplementary file 1 — Supplementary Information [file 41467_2021_21549_MOESM1_ESM.pdf]

SUPPLIMENTARY INFORMATION

Supplementary methods

Antibodies

The antibodies used for this study are: phospho-ERK T202/Y204 (1:1000) (Cell Signaling Technology, #4370), phospho-Rb S807/811 (1:1000) (Cell Signaling Technology, #8516P), GAPDH (1:2000) (Cell Signaling Technology, #5174), ATF4 (1:200) (Cell Signaling Technology, #11815S), phospho-Rb S780 (1:1500) (BD Biosciences, #558385), phospho-S6 S240/244 (1:250) (Cell Signaling Technology, #2215), AXL (1:200) (Cell Signaling Technology, #8661), MITF (1:100) (Abcam, #ab3201), NGFR (1:1000) (Cell Signaling Technology, #8238), SOX10 (1:1000) (Cell Signaling Technology, #89356), FANCD2 (1:500) (Novus Biologicals, #NB100-182), γ-H2AX (1:400) (Cell Signaling Technology, #9718), and MCM2 (1:200) (BD Biosciences, #610700). Secondary antibodies were used at 1:500 dilution (anti-rabbit Alexa Fluor-647: Thermo Fisher, #A-21245; anti-rabbit Alexa Fluor-488: #A-11034; anti-mouse Alexa Fluor-488: #11029; anti-mouse Alexa Fluor-546: #11030); and anti-rabbit IgG, HRP-linked secondary antibody (Cell Signaling Technology, #7074S) for western blotting at 1:1000.

Western blot

Cells were washed 3 times with PBS and lysed in 2x LDS sample buffer (Thermo Fisher, #B0008) supplemented with reducing reagent and 1x phosphatase and protease inhibitor. Lysates were sheared with a 1cc U-100 insulin syringe (Becton Dickinson, #329424) and heated at 95 °C for 10 min. Proteins were separated by Bolt 4-12% Bis-Tris Plus gel (Thermo Fisher, NW04125BOX) and transferred to a PVDF membrane (Merck Millipore, #IPFL00010). The membrane was incubated in 3% BSA (GoldBio, #A-421-250) supplemented with 0.1% Tween-20 (Thermo Fisher, #9005-64-5) at room temperature for 2 hr before overnight incubation with antibodies against phospho-ERK$^{T202/Y204}$ (1:1000), phospho-Rb$^{S807/811}$ (1:1000), and GAPDH (1:2000). The membrane was then washed for 5 min with PBS supplemented with 0.1%

Tween-20 five times and then incubated with anti-rabbit IgG, HRP-linked secondary antibody. The chemiluminescent signals were detected on an Azure C600 from Azure Biosystems.

EdU incorporation assay

To identify cells in S phase of the cell cycle, cells were pulsed with 10 µM EdU at 37 °C for 15 minutes prior to fixation with 4% paraformaldehyde. The EdU was visualized as described in the manufacturer's protocol (Thermo Fisher, #C10340 and #C10641). Cells were then twice washed with PBS and blocked with 3% BSA for 1 hr at room temperature to prepare for further immunostaining.

Apoptosis assay

Cells were seeded in 12-well plates (Corning, #3513), at 10^5 cells/well, 24 hr prior to drug treatments. Wells were treated in triplicate with various doses and combinations of MAPK pathway inhibitors used in the study for 2 days, 4 days, or 2 weeks. Etoposide (10 µM) was used as a positive control for apoptosis. After treatment, non-adherent cells were first harvested by pipetting, adherent cells were harvested by trypsinization, and these two populations were then combined. Cell suspensions were centrifuged and resuspended in calcium-rich binding buffer provided by the apoptosis staining kit (abcam, #ab14085) to reach ~10^6 cells/mL. Live suspensions were stained with both Annexin V-FITC (1:100) and propidium iodide, PI (1:100). Single-cell fluorescent signals were acquired on a BD FACSCelesta flow cytometer equipped with 488 and 561 nm lasers. By convention, cells were gated in FlowJo to remove debris and doublets. Annexin V-FITC and PI values were plotted as a bivariate scatter and etoposide-determined quadrant gating was applied to all plots to reach final apoptotic population percentages.

siRNA transfection

siRNA transfections were performed using the DharmaFECT 4 reagent (Dharmacon, #T-2004-02) according to the manufacturer's instructions. The transfection mix was added to the cells at the time of drug treatment and removed after 6 hr. The knockdown efficiency was determined by RNA FISH 72 hr post-transfection. Oligonucleotides used in this study are: DS NC-1 (IDT, #51-01-14-04), *LINC01133* DsiRNA

(IDT, #hs.Ri.LINC01133.13.1, #hs.Ri.LINC01133.13.2, #hs.Ri.LINC01133.13.3), *ATF4* DsiRNA (IDT, #hs.Ri.ATF4.13.3, #hs.Ri.ATF4.13.1), *RAB32* DsiRNA (IDT, #hs.Ri.RAB32.13.1, #hs.Ri.RAB32.13.2, #hs.Ri.RAB32.13.3), *CDC42EP1* DsiRNA (IDT, #hs.Ri.CDC42EP1.13.1, #hs.Ri.CDC42EP1.13.2, #hs.Ri.CDC42EP1.13.3)

Dose-response curve fitting

Dose-response curve fits for each cell line's pRb^+ percentage after dabrafenib were calculated using GraphPad Prism (v8.3). All cell line dose-response curves were fit using the following standard inhibitory Hill function.

$$f(c) = f(\infty) + \frac{f(0) - f(\infty)}{1 + \left(\frac{c}{\mathrm{IC}_{50}}\right)^n} \quad (1)$$

Here, $f(c)$ is the fraction of proliferative cells (pRb^+) in the population at drug concentration c; $f(0)$ and $f(\infty)$ are the fractions at no drug and at the maximal tested drug concentration, respectively; IC_{50} is the half-maximal inhibitory concentration of the drug; and n is the Hill coefficient. $f(c)$, $f(0)$, and $f(\infty)$ were obtained from experiments; and the values of IC_{50} and n were obtained from fitting. The biphasic shape of the A375 dose-response curves was fitted by the summation of two sigmoidal inhibitory curves (employed in GraphPad Prism as a biphasic fit function).

a

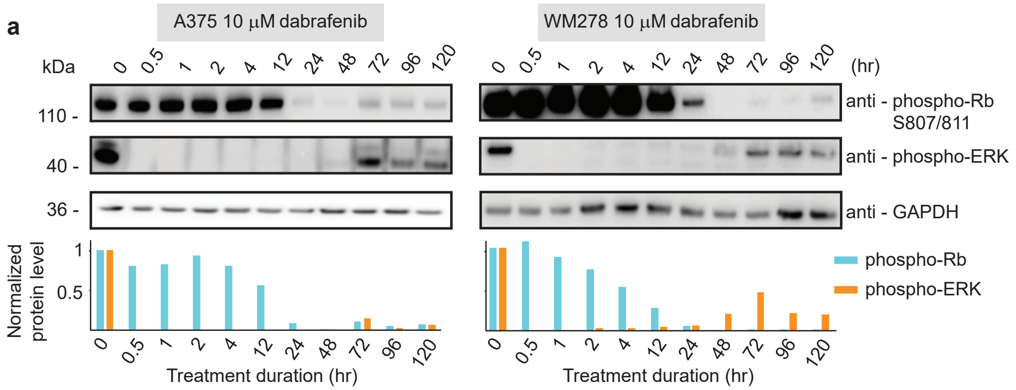

b

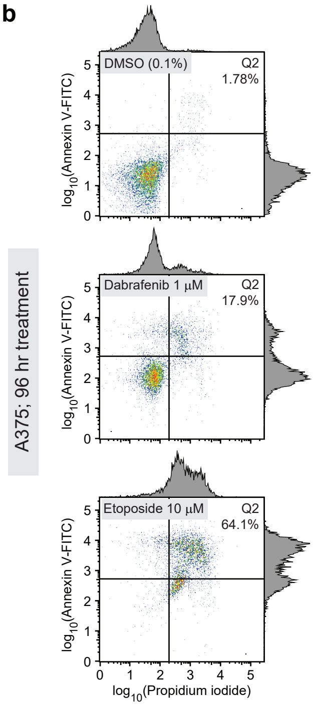

c

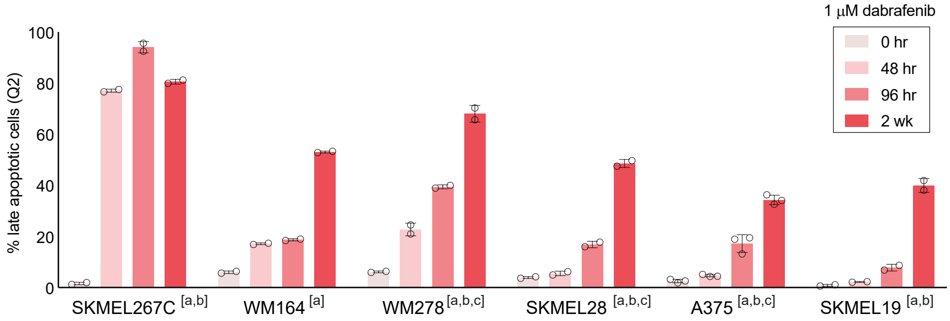

	SKMEL267C [a,b]	WM164 [a]	WM278 [a,b,c]	SKMEL28 [a,b,c]	A375 [a,b,c]	SKMEL19 [a,b]
BRAF	V600E/+	V600E/+	V600E/+	V600E/V600E	V600E/V600E	V600E/V600E
PTEN	WT	WT	-/+	T167A/T167A	WT	WT
EGFR	WT	WT	WT	P753S/P753S	WT	WT
TP53	WT	WT	WT	L145R/L145R	WT	N131K
CDK4	WT	WT	WT	R24C/+	WT	WT
CDKN2A	WT	WT	WT	WT	E61*/E61*	-/-

Cell line genotyping sources:

[a] Cellosaurus (https://doi.org/10.7171/jbt.18-2902-002)

[b] COSMIC: The Catalog of Somatic Mutations in Cancer (https://doi.org/10.1093/nar/gky1015)

[c] Cancer Cell Line Encyclopedia (https://doi.org/10.1038/nature11003)

Supplementary Figure 1

Supplementary Figure 1 | Dabrafenib treatment induces apoptosis to varying degrees in different cell lines, while surviving cells experience a rebound in MAPK signalling and cell proliferation. a, A375 (left) and WM278 (right) cells were treated with 10 μM dabrafenib for the indicated durations and the levels of phospho-ERK and phospho-Rb S807/811 were measured by western blot (top). The western blot signals were quantified by normalizing first to GAPDH levels, then to the untreated condition (bottom) (n=1). **b-c**, Apoptotic cell quantification by flow cytometric analyses of Annexin V-FITC and propidium iodide staining. DMSO bivariate plot is shown as a negative control for apoptosis; etoposide is shown as a positive control. Representative bivariate plot is shown for 96 hr of 1 μM dabrafenib (b). Quantified replicates of late apoptotic cells (Q2) in each treatment condition are shown for the indicated time points for six different melanoma cell lines (c). Error bars: mean ± std of at least 2 biological replicates, representative of 2 experimental repeats. Genotype for each cell line is listed. Source data are provided as a Source Data file.

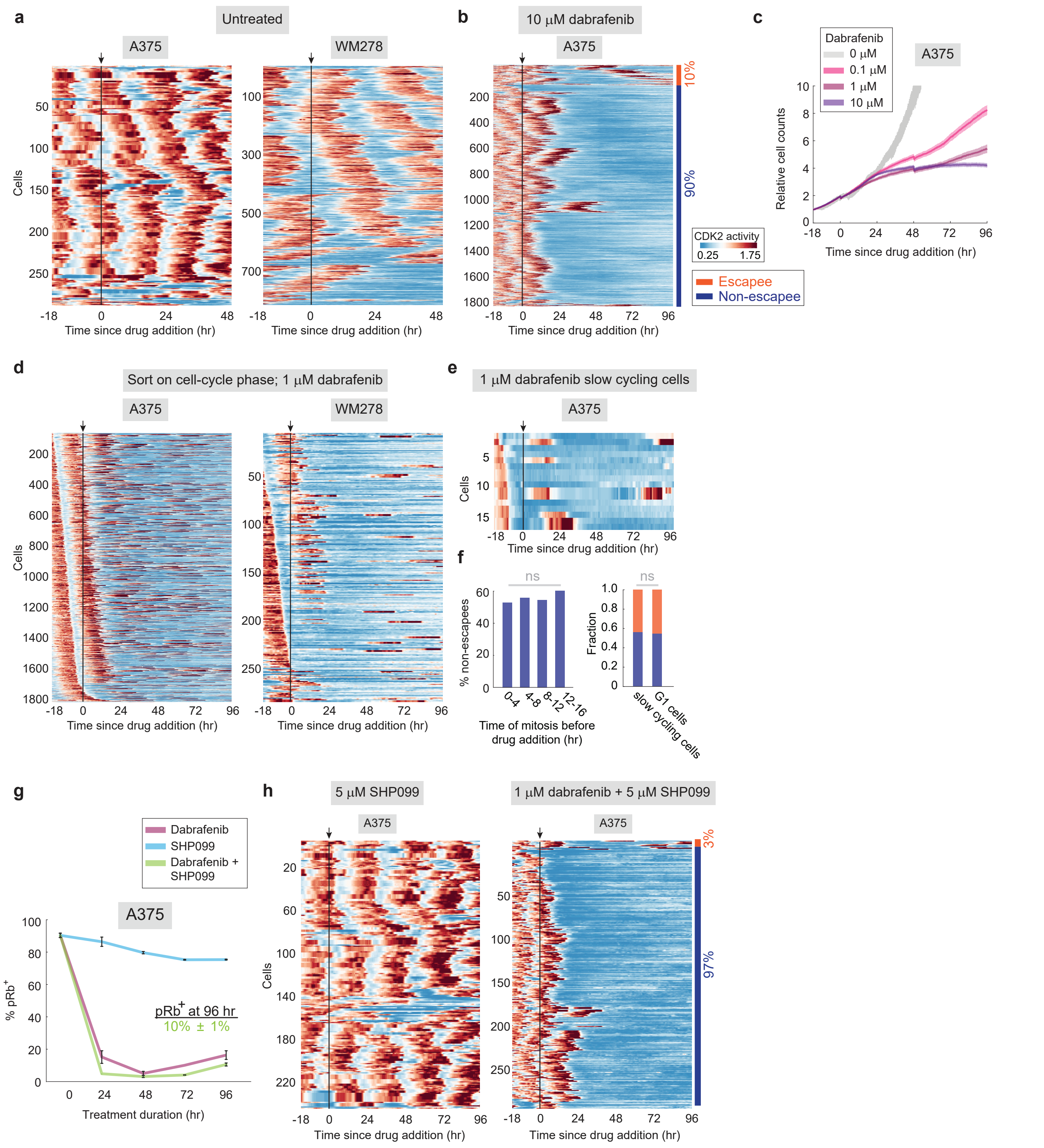

Supplementary Figure 2

Supplementary Figure 2 | Cellular heterogeneity in drug response is revealed by live-cell imaging. a-b, Heatmap of single-cell CDK2 activity traces in untreated A375 and WM278 cells or 10 µM dabrafenib-treated A375 cells. The black line marks the time of media change or drug addition. The percentages mark the proportion of cells with each behaviour. **c**, Relative cell count over time in A375 cells treated with dabrafenib, as monitored by time-lapse microscopy. Error bars: 95% confidence interval. n ≥ 20 biologically independent samples. **d**, Replotting of data in Fig. 2c by sorting traces according to how long ago cells underwent mitosis prior to drug addition. **e**, Heatmap of CDK2 activity for 16 slow-cycling cells sorted according to how long ago cells underwent mitosis prior to drug addition, showing that these cells can still readily escape drug. Cells were designated slow-cycling if they were in a $CDK2^{low}$ quiescence for more than 6 hr prior to drug addition. **f**, Left: fraction of non-escapees in different cell-cycle phases at the time of 1 µM dabrafenib addition. Cells were classified into different cell-cycle phases based on the time of mitosis before drug treatment. Then the fraction of non-escapees was calculated based on the cell behaviour over the 96 hr drug treatment duration. For each category, a binomial test was performed to evaluate the difference between the reported fraction and the fraction in all cells (57%, Fig. 2c). Right: percentage of escapees and non-escapees between naturally slow-cycling cells and G1 cells. Cells were designated as G1 cells if CDK2 activity rose no more than 6 hr prior to drug addition; 625 cells met this criterion. A binomial test was performed to compare the fraction of non-escapees in the two categories. **g**. Quantification of percentage of pRb^+ cells in A375 cells treated for the indicated duration with 1 µM dabrafenib or 5 µM SHP099 or in combination. The percentage of pRb^+ cells under the combined treatment at 96 hr is noted. Error bars: mean ± std of 3 replicate wells. **h**. Heatmap of A375 single-cell CDK2 activity traces in 5 µM SHP099 or combination of dabrafenib and SHP099. Source data are provided as a Source Data file.

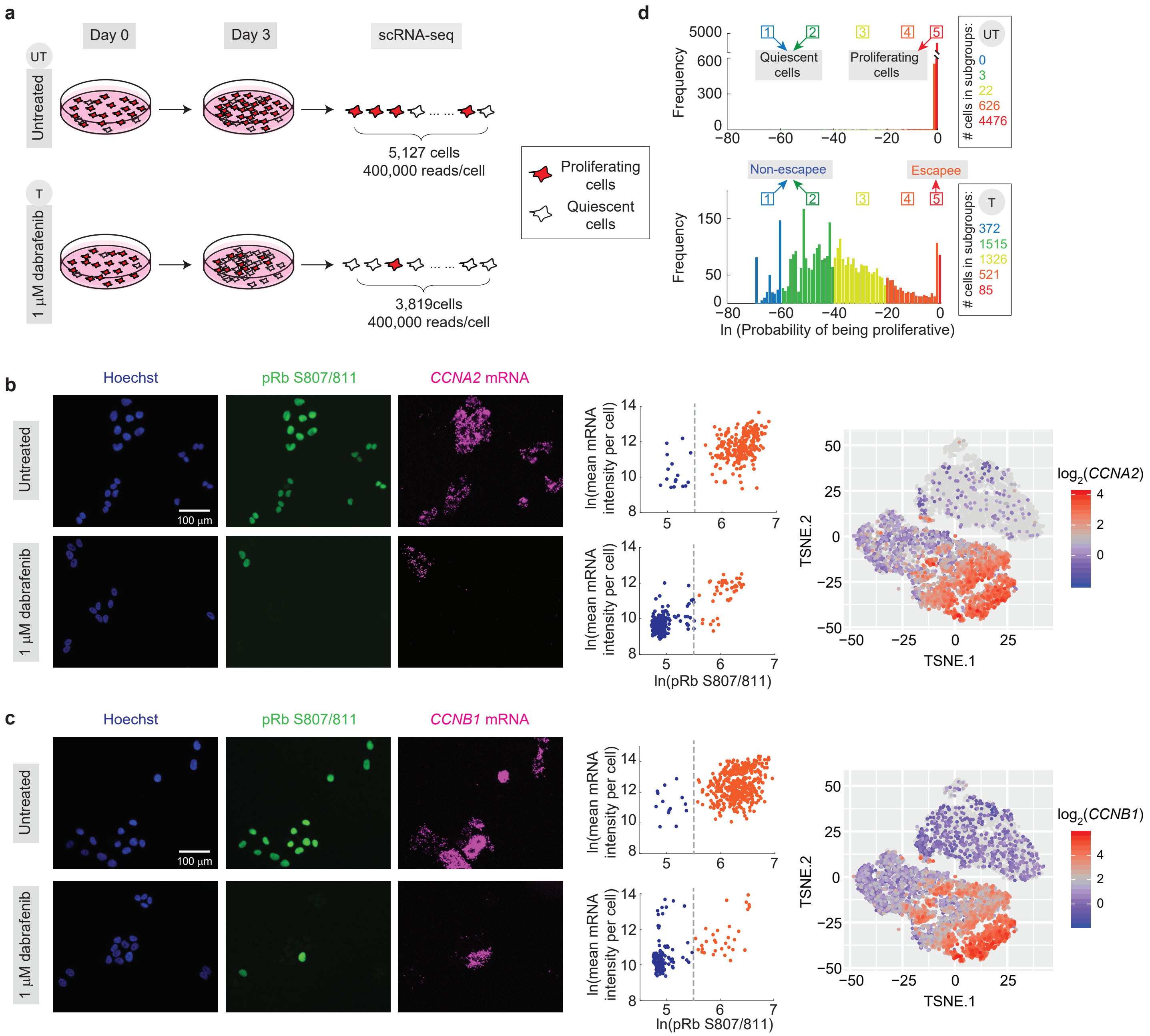

Supplementary Figure 3

Supplementary Figure 3 | Calculation of a cell's proliferation probability based on 51 cell-cycle genes, and validation of two of the genes. a, Schematic of scRNA-seq experiment. **b-c**, mRNA expression levels of *CCNA2* and *CCNB1* in untreated A375 cells or in cells treated with 1 μM dabrafenib for 72 hr. Left: co-staining of phospho-Rb and the indicated mRNA; Middle: quantification of mRNA levels in pRb^+ and pRb^- cells (each population pooled from 2 replicate wells); Right: tSNE plots based on scRNA-seq data showing increased expression of these two genes in the escapee subpopulation, visible as a small peninsula of red-shaded cells in the treated population. **d**, Histograms of single-cell proliferation probability in untreated A375 cells (UT, top) and cells treated with 1 μM dabrafenib for 72 hr (T, bottom) conditions. Cells were placed into five categories based on their probability of proliferation (see Methods and Supplementary Data File 1). The number of cells in each category is shown on the right with colours matching their categories.

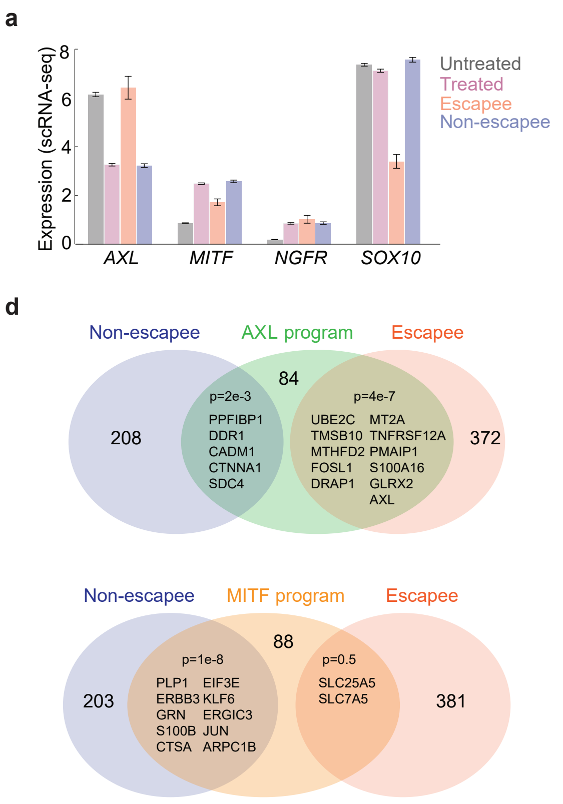

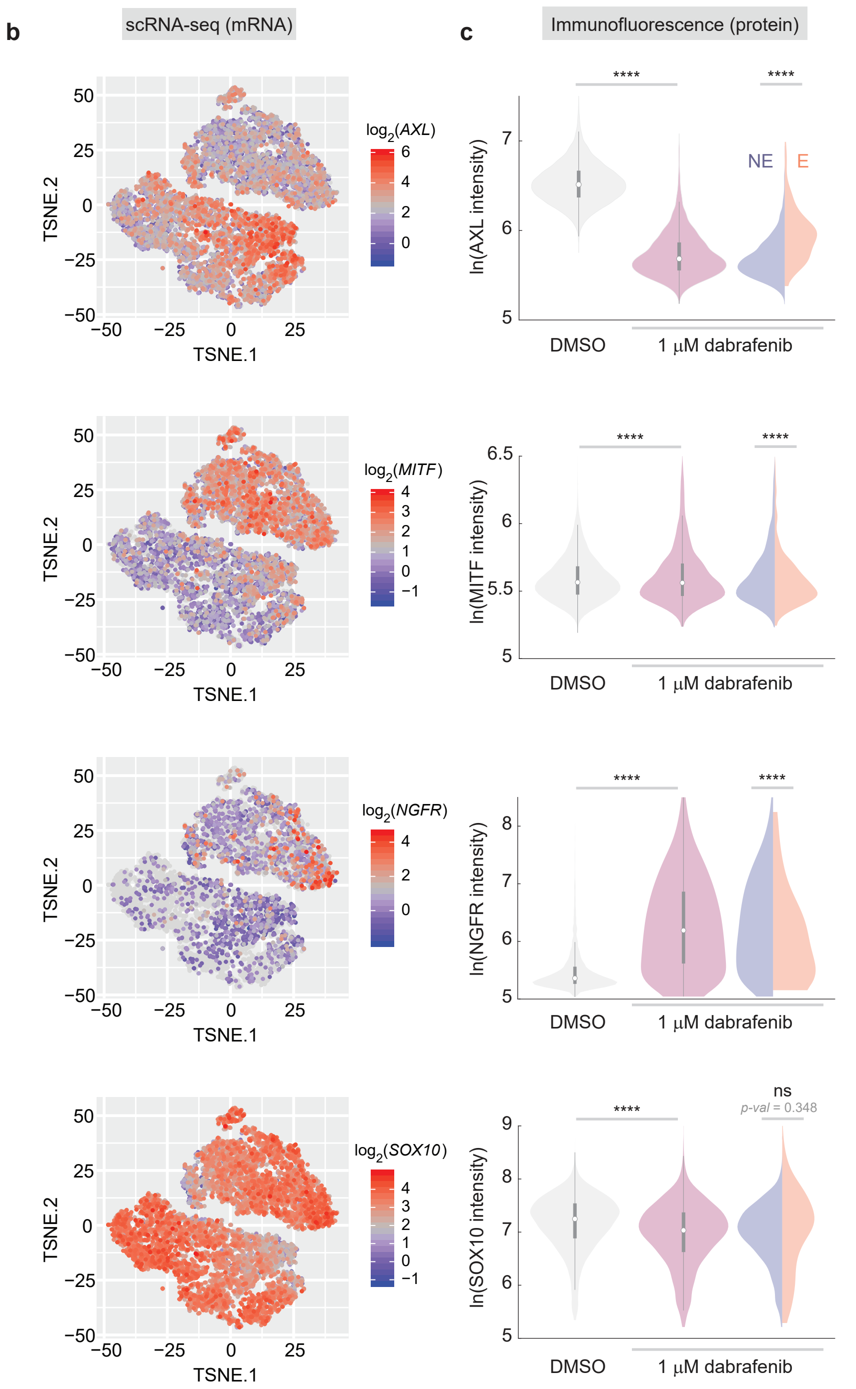

e

p<0.001 p<0.01 p<0.05

		Genes upregulated in: Escapee	Genes upregulated in: Non-escapee	40 intersect genes
Tirosh	MITF program	0.5	1e-8	0.1
	AXL program	4e-7	2e-3	1e-2
Rambow	Pigmentation	1	0.1	1
	Hypometabolic / starved-like	3e-6	1	4e-2
	Invasion	7e-3	8e-3	8e-2
	Neural Crest Stem Cell	1	0.3	1
Tsoi	Undifferentiated	2e-3	1	0.2
	Undiff.-Neural Crest	7e-7	6e-2	2e-5
	Neural Crest-Like	1	2e-3	1
	Neural Crest-Transitory	1	0.2	1
	Transitory	1	3e-2	1
	Transitory-Melanocytic	0.6	0.7	1
	Melanocytic	0.6	0.4	9e-2

Supplementary Figure 4

Supplementary Figure 4 | Escapees show an *AXL*^{high}/*MITF*^{low} gene signature. a, mRNA expression from scRNA-seq for *AXL, MITF, NGFR,* and *SOX10* in A375 cells. Untreated cells (n = 5127): all cells in untreated condition; Treated cells (n = 3819): all cells in treated condition; escapees (n = 85): subgroup 5 in treated condition; non-escapees (n = 1887): subgroup 1 and 2 in treated condition. Bar plot indicates the mean ± SEM, where the SEM reflects technical noise and cell-to-cell variability. **b**, Visualization of single-cell *AXL, MITF, NGFR,* and *SOX10* mRNA expression levels on the combined untreated and treated t-SNE plot. **c**, Violin plot showing the AXL, MITF, NGFR, and SOX10 immunofluorescence signal in A375 cells treated with DMSO or 1 µM dabrafenib for 72 hr. Split violin plot indicates the protein level in dabrafenib-treated escapees (E) and non-escapees (NE), determined by co-staining with phospho-Rb (S807/811) in the case of MITF or phospho-Rb (S780) for the other markers. Each population value is pooled from 3 replicate wells. **d**, Venn diagram showing the overlap of upregulated genes in escapees (subgroup 5 in treated condition) or non-escapees (subgroup 1 and 2 in treated condition) with the *AXL* or *MITF* program published in Tirosh *et al*[1]. Genes upregulated in escapees, n = 383; genes upregulated in non-escapees, n = 213; *AXL*-program: n = 100; *MITF*-program: n = 100. *p* values were computed with the R package 'GeneOverlap[2]' (Methods). **e**, Comparison of upregulated genes in escapees or non-escapees with existing gene signatures. Comparison with Tirosh *et al*[1]. was reproduced from (d). Sizes of gene signatures in Rambow *et al.*[3]: pigmentation, n = 15; hypometabolic/starved-like, n = 27; invasion, n = 49; neural crest stem cell, n = 37. Sizes of gene signatures in Tsoi *et al.*[4]: undifferentiation, n = 118; undiff.-neural crest, n = 106; neural crest-like, n = 66; neural crest-transitory, n = 25; transitory, n = 29; transitory-melanocytic, n = 125; melanocytic, n = 62. Source data are provided as a Source Data file.

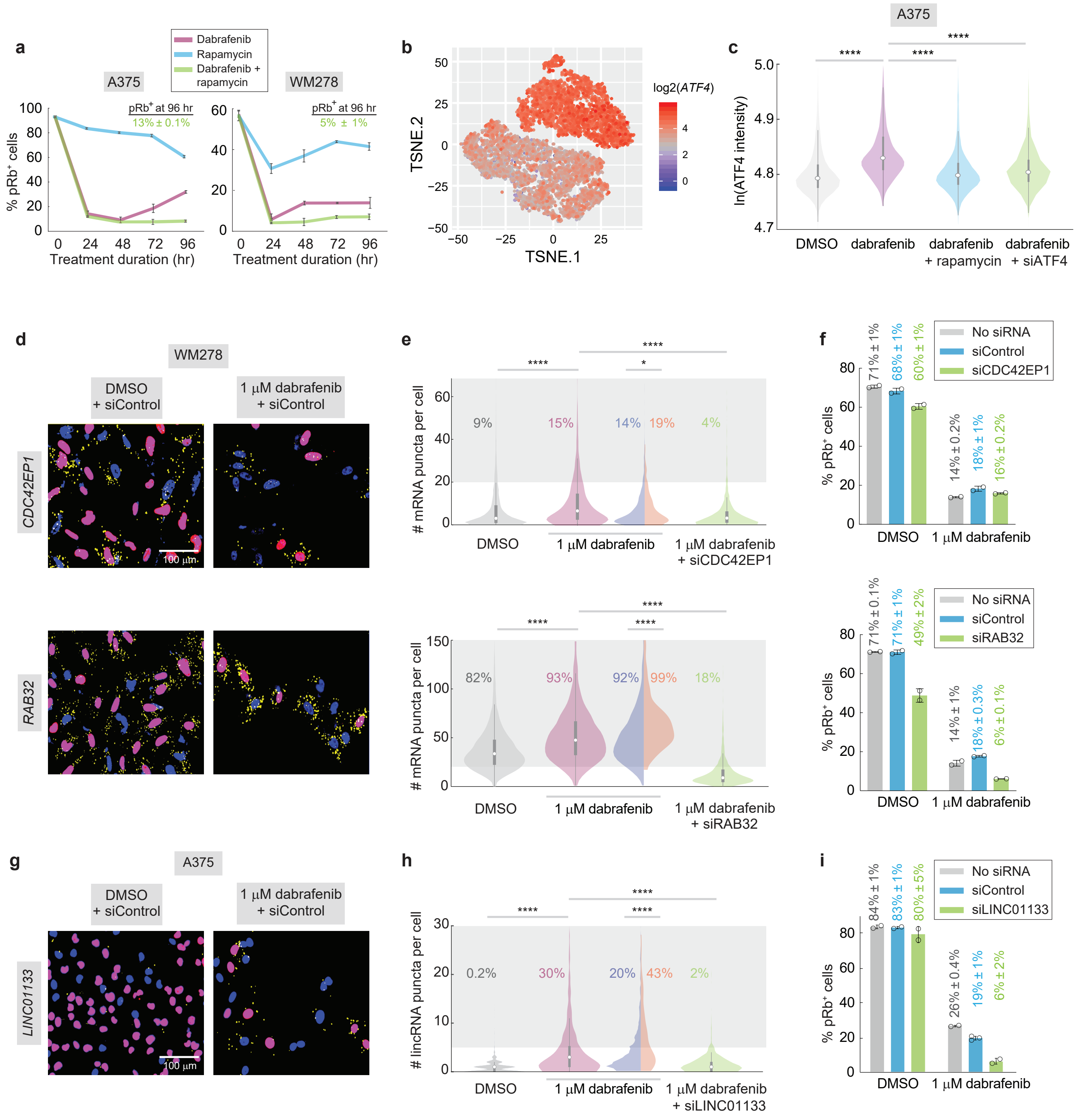

Supplementary Figure 5

Supplementary Figure 5 | ATF4 target genes promote escape from dabrafenib in A375 and WM278 cells. a, Percentage of pRb^+ cells in A375 or WM278 cells treated for indicated durations with 1 µM dabrafenib or 10 nM rapamycin alone, or in combination. Error bars: mean ± std of 3 replicate wells. **b,** Visualization of single-cell ATF4 mRNA levels on the combined t-SNE plot, showing homogenous upregulation of ATF4 mRNA in all treated cells. **c**, Violin plots showing ATF4 protein levels by immunofluorescence in A375 cells treated as indicated for 72 hr. ATF4 expression is significantly induced by dabrafenib but repressed by addition of the mTORC1 inhibitor rapamycin or siATF4. Each population value is pooled from 2 replicate wells. **d,** Representative images of WM278 cells stained for *CDC42EP1* or *RAB32* mRNA (yellow), phospho-Rb (pink), and Hoechst (blue), for the indicated 72 hr treatment conditions. **e**, Quantification of number of mRNA puncta in WM278 cells for *CDC42EP1* (upper panel) and *RAB32* (lower panel) in each condition indicated. The percentage of cells that have > 20 mRNA puncta for *CDC42EP1* or *RAB32* are indicated on the plot of each condition. Each population value is pooled from 2 replicate wells. **f**, The percentage of pRb^+ WM278 cells after 72 hr treatment with DMSO or 1 µM dabrafenib and either no siRNA (grey), control siRNA (blue) or siRNA against *CDC42EP1* (upper panel) or *RAB32* (lower panel) (green). Error bars: mean ± std of 2 biological replicates (two wells pooled per data point), representative of 2 experimental repeats. **g-i**, Same analysis as (**d-f**), but for *LINC01133* in A375 cells. The percentage of cells that have > 5 mRNA puncta for *LINC01133* is indicated on the plot of each condition. For the DMSO-treated violin plot in (h), most cells have zero puncta and only a few cells have low integer numbers of puncta, thus the violin plot appears staggered at the integer values. Source data are provided as a Source Data file.

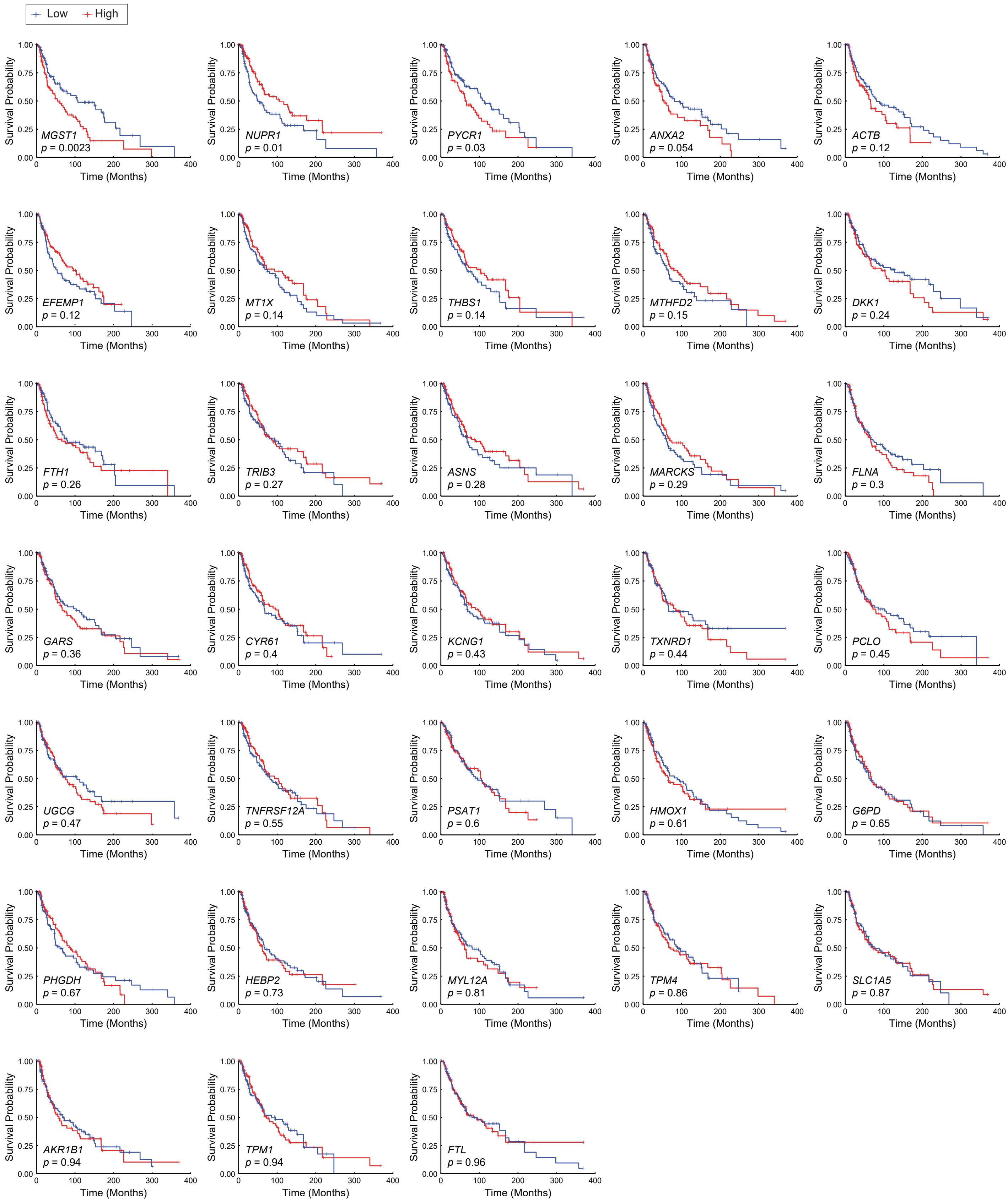

Supplementary Figure 6

Supplementary Figure 6 | Melanoma patient survival curves for 33 of 40 upregulated genes in escapees that do not show significantly worse correlation. Melanoma patient survival curves for 33 of 40 uniquely upregulated genes in escapees. *p* value is indicated on each plot. *p* value: log-rank test.

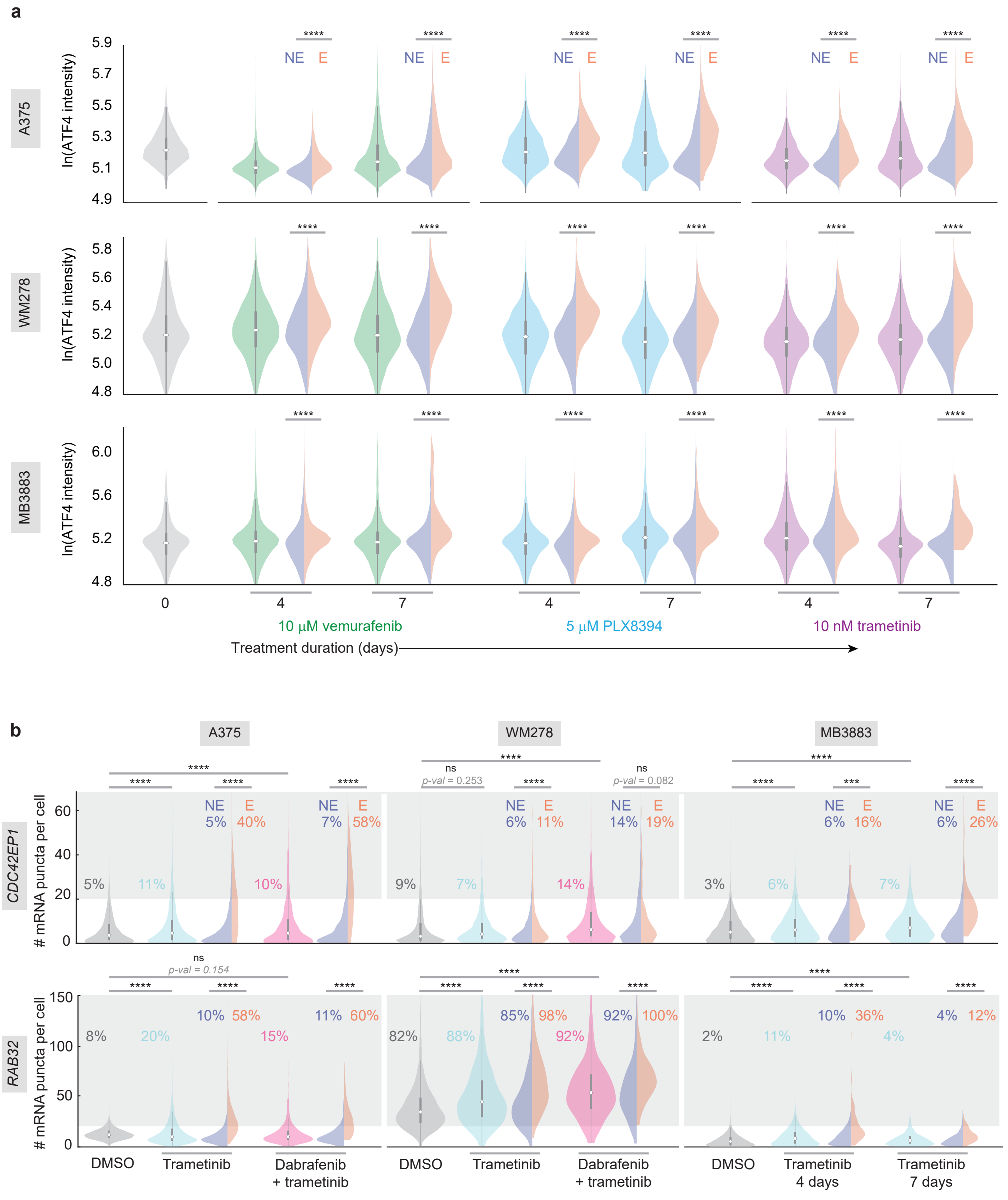

Supplementary Figure 7

Supplementary Figure 7 | The escapee phenotype is observed with multiple MAPK pathway inhibitors and also occurs in *ex vivo* patient biopsies. a, Violin plots of nuclear ATF4 protein intensity in A375, WM278, and MB3883 cells treated with the indicated drugs for 0, 4, or 7 days. Each population value is pooled from 4 replicate wells. **b**, Violin plots showing the number of *CDC42EP1*or *RAB32* mRNA puncta in A375 and WM278 cells treated with DMSO, 10 nM trametinib, or 1 μM dabrafenib plus 10 nM trametinib for 72 hr. Far-right section shows the number of *CDC42EP1* or *RAB32* mRNA puncta in MB3883 cells treated for 0, 4 or 7 days with 1 nM trametinib. The percentage of cells that have > 20 mRNA puncta for each gene is indicated on the plot. Each population value is pooled from 2 replicate wells.

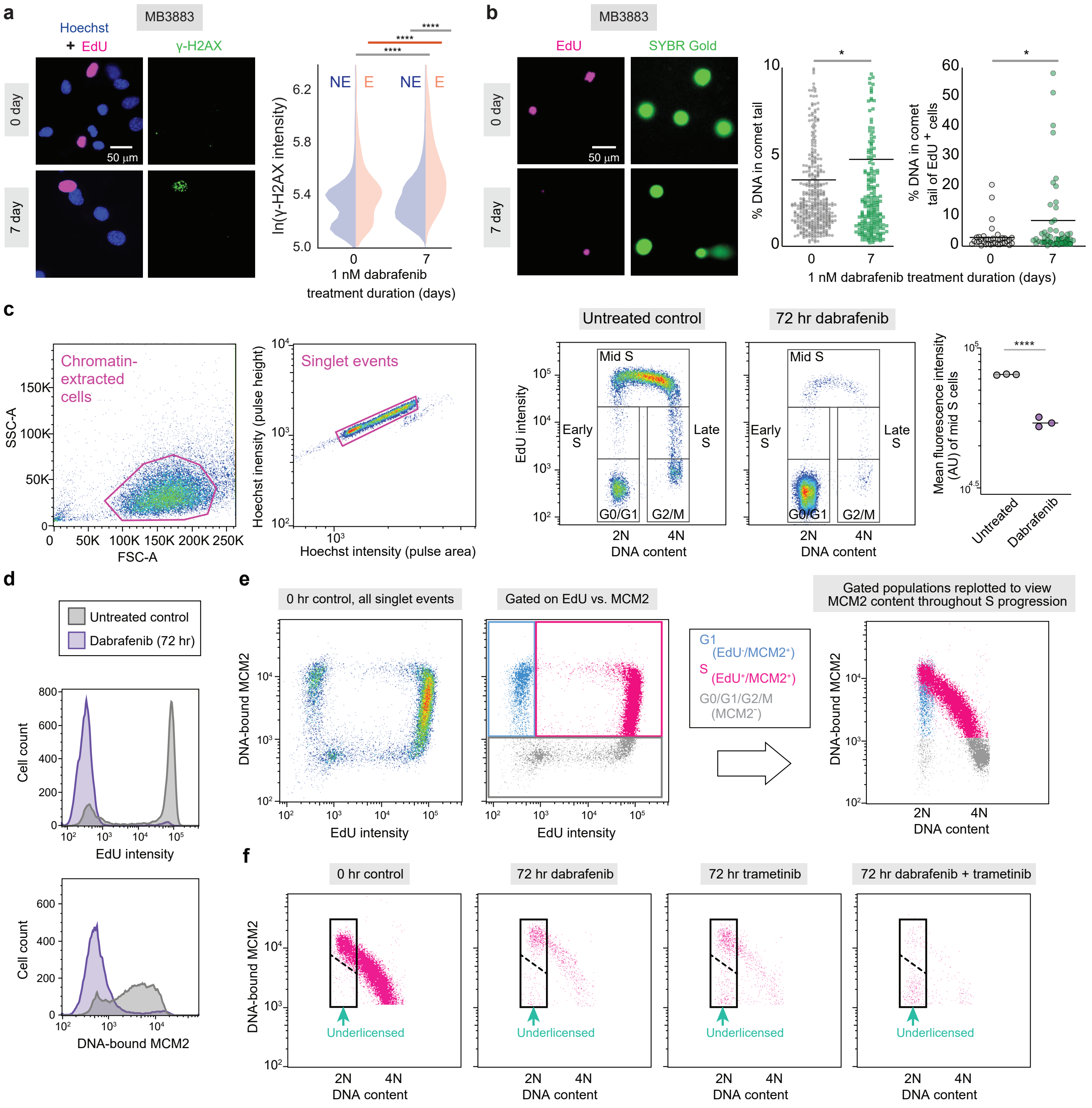
a
MB3883
Hoechst
+ EdU
γ-H2AX
0 day
7 day
50 µm
NE
E
ln(γ-H2AX intensity)
1 nM dabrafenib
treatment duration (days)
b
MB3883
EdU
SYBR Gold
% DNA in comet tail
% DNA in comet tail of EdU + cells
1 nM dabrafenib treatment duration (days)
c
Chromatin-extracted cells
SSC-A
FSC-A
Singlet events
Hoechst inensity (pulse height)
Hoechst intensity (pulse area)
Untreated control
72 hr dabrafenib
Mid S
Early S
Late S
G0/G1
G2/M
EdU intensity
DNA content
Mean fluorescence intensity (AU) of mid S cells
Untreated
Dabrafenib
d
Untreated control
Dabrafenib (72 hr)
Cell count
EdU intensity
DNA-bound MCM2
e
0 hr control, all singlet events
Gated on EdU vs. MCM2
G1 (EdU⁻/MCM2⁺)
S (EdU⁺/MCM2⁺)
G0/G1/G2/M (MCM2⁻)
Gated populations replotted to view MCM2 content throughout S progression
DNA-bound MCM2
EdU intensity
DNA content
f
0 hr control
72 hr dabrafenib
72 hr trametinib
72 hr dabrafenib + trametinib
Underlicensed
DNA content

Supplementary Figure 8 | Significant DNA damage is observed in escapees detected in *ex vivo* patient cultures, and cells cycling in the presence of dabrafenib and trametinib show aberrant licensing of origins of replication. a, Representative images of 1 nM dabrafenib-treated MB3883 patient cells stained for EdU incorporation and γ-H2AX. Quantifications of the γ-H2AX puncta for escapees (E) and non-escapees (NE) are plotted as split violins. Each population value is pooled from 6 replicate wells. **b,** Neutral comet assay analysis in 1 nM dabrafenib-treated MB3883 patient cells. Images show gels co-stained for SYBR Gold to mark DNA and EdU to mark cells in S phase. Plots show all cells (left) or only EdU^+ cells (right) in which the percent of DNA in each comet tail was measured, with mean values indicated on the plots as horizontal lines. Each population value is pooled from 2 biological comet slide replicates. Left panel: 0 and 7 days, n = 398, 299 biologically independent cells. Right panel: 0 and 7 days, n = 37, 45 biologically independent cells. **c,** Gating scheme to identify single chromatin-extracted cells by flow cytometry (n = 3 biologically independent experiments). Scatter plot of DNA synthesis rate (EdU incorporation, 30 min pulse) vs. DNA content in untreated A375 cells to identify cell-cycle phases. The same gates are propagated to the plot of dabrafenib-treated cells. Right-most plot shows mean fluorescence intensity of all cells in mid S phase (3 replicate samples). **d,** Representative histograms of DNA synthesis rate (EdU intensity) and chromatin-associated MCM2 for untreated and dabrafenib-treated cells. **e,** Gating scheme (as in Mantson *et al.*[5]) used to identify cell-cycle phases by EdU and chromatin-bound MCM2 signal intensity in untreated cells. Cells that are both EdU^+ and chromatin-bound $MCM2^+$ are shaded pink. **f,** Scatter plot of EdU^+ and chromatin-bound $MCM2^+$ cells gated as in (e) for the indicated treatment conditions (pooled from 3 replicate samples). Untreated cells showing normal licensing of replication origins are used to draw the gate (left-most plot), and this gate is then propagated to the treated conditions. Cells falling below the dashed line are under-licensed. The percentage of under-licensed cells out of all early S cells (entire rectangle) is reported in Fig. 7d for 3 replicates. Source data are provided as a Source Data file.

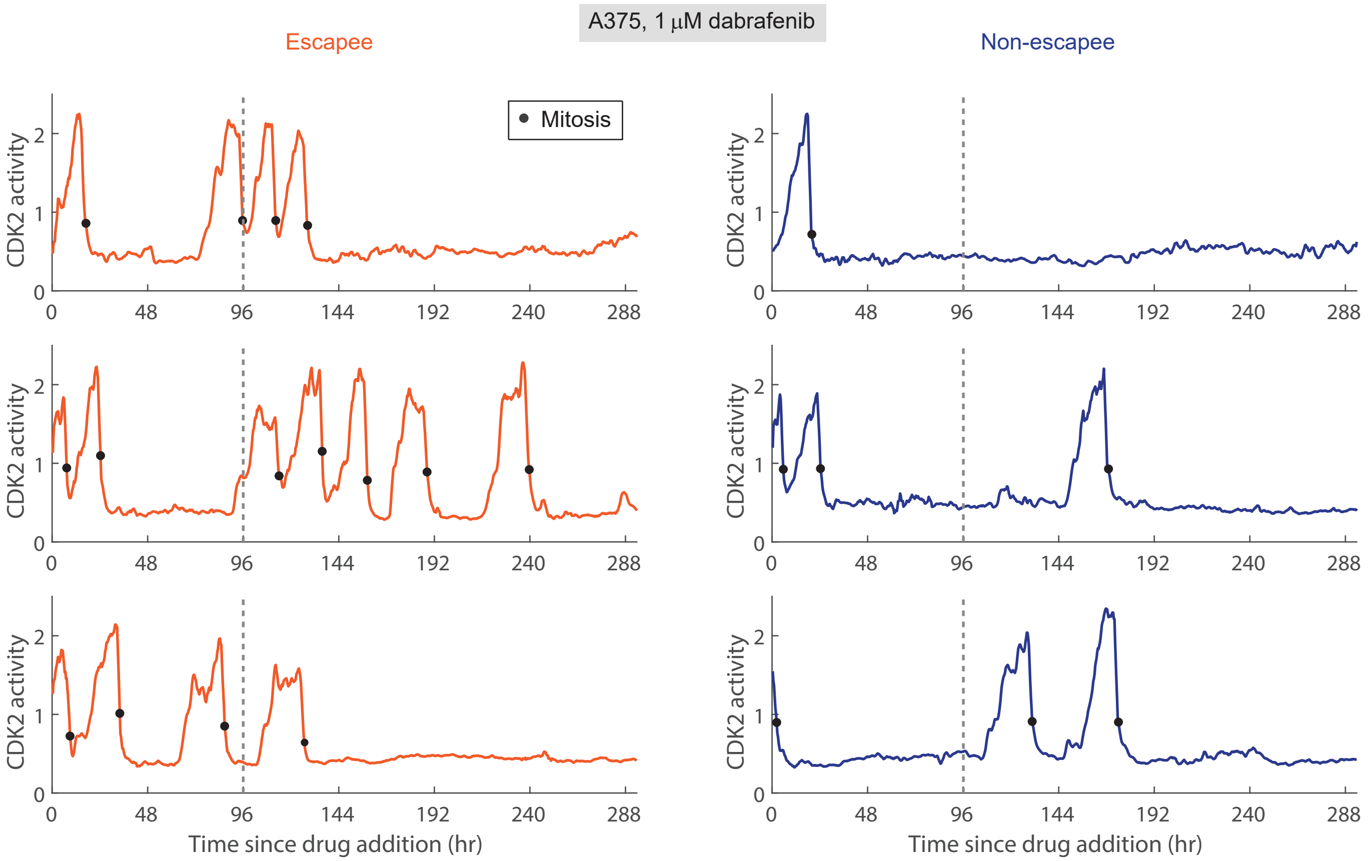

Supplementary Figure 9

Supplementary Figure 9 | Escapees outgrow non-escapees over extended treatment. Sample single-cell CDK2 activity traces from a 12-day movie of A375 cells treated with 1 µM dabrafenib at the start of the movie. Escapees and non-escapees are defined by their behavior during the first 96 hr of filming (dashed line). Black dots mark mitoses.

Supplementary References

1. Tirosh, I. *et al.* Dissecting the multicellular ecosystem of metastatic melanoma by single-cell RNA-seq. *Science* **352**, 189–196 (2016).
2. Sinai, L. S., Mount. *GeneOverlap*. (Bioconductor, 2017). doi:10.18129/B9.BIOC.GENEOVERLAP.
3. Rambow, F. *et al.* Toward Minimal Residual Disease-Directed Therapy in Melanoma. *Cell* **174**, 843-855.e19 (2018).
4. Tsoi, J. *et al.* Multi-stage Differentiation Defines Melanoma Subtypes with Differential Vulnerability to Drug-Induced Iron-Dependent Oxidative Stress. *Cancer Cell* **33**, 890-904.e5 (2018).
5. Matson, J. P. *et al.* Intrinsic checkpoint deficiency during cell cycle re-entry from quiescence. *J. Cell Biol.* **218**, 2169–2184 (2019).
