# Supplementary material for: Melanoma subpopulations that rapidly escape MAPK pathway inhibition incur DNA damage and rely on stress signalling
Source: Nat Commun. 2021 Mar 19;12:1747. doi: 10.1038/s41467-021-21549-x (PMC7979728; doi:10.1038/s41467-021-21549-x)
Supplement: Supplementary file 3 — Description of Additional Supplementary Files [file 41467_2021_21549_MOESM3_ESM.pdf]

Description of Additional Supplementary Files

File Name: Supplementary Data 1
Description: Tab 1: List of 51 cell-cycle genes used to calculate proliferation probability; Tab 2-3: List of 40 significantly upregulated genes and 16 significantly downregulated genes in escapees.

File Name: Supplementary Data 2
Description: Hazard ratios and p-values of the Cox proportional hazards analysis. Positive hazard ratios indicate negative correlations with patient survival. Significant genes ($p < 0.05$) are highlighted in bold. *LINC01133* and *TMSB4X* are excluded due to the absence of measurements in the TCGA datasets.

File Name: Supplementary Movie 1
Description: **CDK2 activity in untreated A375 melanoma cells.** A375 cells expressing DHB-mCherry were cultured in phenol-red free full growth media. The CDK2 activity trace for the cell with the yellow arrow is plotted underneath. Red dots mark mitosis.

File Name: Supplementary Movie 2
Description: **CDK2 activity in 10 µM dabrafenib-treated A375 cells.** A375 cells expressing DHB-mCherry were treated with 10 µM dabrafenib at the start of the movie. Two different cell behaviors (escapee and non-escapee) are sequentially displayed in this movie: the cell with a yellow arrow is a non-escapee and the corresponding CDK2 activity trace is plotted below in black; the cell with the red arrow is an escapee and the corresponding CDK2 activity trace is plotted below in red. Red dots mark mitosis.

File Name: Supplementary Movie 3
Description: **CDK2 activity in monotherapy or combination therapy-treated A375 cells. a**, Untreated A375 cells. **b**, 1 µM dabrafenib-treated A375 cells. **c**, 10 nM trametinib-treated A375 cells. **d**, A375 cells treated with 1 µM dabrafenib plus 10 nM trametinib. Cells were imaged in full-growth media for 18 hr before drug addition; the movie was then paused for drug addition and imaging continued for 96 hr with a pause at 48 hr for drug refreshment. Flash indicates time of drug addition.

File Name: Supplementary Movie 4
Description: **CDK2 activity in monotherapy or combination therapy-treated WM278 cells. a**, Untreated MW278 cells. **b**, 1 µM dabrafenib-treated WM278 cells. **c**, 10 nM trametinib-treated WM278 cells. **d**, WM278 cells treated with 1 µM dabrafenib plus 10 nM trametinib. Cells were imaged in full-growth media for 18 hr before drug addition; the movie was then paused for drug addition and imaging continued for 96 hr with a pause at 48 hr for drug refreshment. Flash indicates time of drug addition.
