# Supplementary material for: Melanoma subpopulations that rapidly escape MAPK pathway inhibition incur DNA damage and rely on stress signalling
Source: Nat Commun. 2021 Mar 19;12:1747. doi: 10.1038/s41467-021-21549-x (PMC7979728; doi:10.1038/s41467-021-21549-x)
Supplement: Supplementary file 10 — Reporting Summary [file 41467_2021_21549_MOESM10_ESM.pdf]

nature research

Corresponding author(s): Sanrina L. Spencer

Last updated by author(s): Jan 11, 2021

Reporting Summary

Nature Research wishes to improve the reproducibility of the work that we publish. This form provides structure for consistency and transparency in reporting. For further information on Nature Research policies, see our Editorial Policies and the Editorial Policy Checklist.

Statistics

For all statistical analyses, confirm that the following items are present in the figure legend, table legend, main text, or Methods section.

n/a	Confirmed	
☐	☒	The exact sample size (n) for each experimental group/condition, given as a discrete number and unit of measurement
☐	☒	A statement on whether measurements were taken from distinct samples or whether the same sample was measured repeatedly
☐	☒	The statistical test(s) used AND whether they are one- or two-sided<br>*Only common tests should be described solely by name; describe more complex techniques in the Methods section.*
☐	☒	A description of all covariates tested
☐	☒	A description of any assumptions or corrections, such as tests of normality and adjustment for multiple comparisons
☐	☒	A full description of the statistical parameters including central tendency (e.g. means) or other basic estimates (e.g. regression coefficient) AND variation (e.g. standard deviation) or associated estimates of uncertainty (e.g. confidence intervals)
☐	☒	For null hypothesis testing, the test statistic (e.g. F, t, r) with confidence intervals, effect sizes, degrees of freedom and P value noted<br>*Give P values as exact values whenever suitable.*
☐	☒	For Bayesian analysis, information on the choice of priors and Markov chain Monte Carlo settings
☐	☒	For hierarchical and complex designs, identification of the appropriate level for tests and full reporting of outcomes
☒	☐	Estimates of effect sizes (e.g. Cohen's d, Pearson's r), indicating how they were calculated

Our web collection on statistics for biologists contains articles on many of the points above.

Software and code

Policy information about availability of computer code

Data collection	Nikon NIS Elements 5.11.02<br>PerKinElmer Opera Phenix HH14000000<br>FACSDiva BD Biosciences 8.0.1.1
Data analysis	ImageJ 1.52e<br>MATLAB Mathworks 2017a<br>R The R Foundation 3.5.0<br>RStudio Server RStudio 1.1.383<br>GraphPad Prism GraphPad Software 8.3<br>FlowJo FlowJo LLC 10.6.1<br>Cell Ranger 10X Genomics 2.0.2<br>Cell Ranger R Kit 10X Genomics 2.0.0<br>Seurat Rahul Satija Lab 3.1.0<br>GeneOverlap Li Shen 1.18.0<br>Cytoscape Cytoscape Consortium 3.7.1<br>iRegulon KU Leuven 1.3<br>OncoLnc OmnesRes N/A<br>Harmony high-content imaging and analysis software PerkinElmer HH17000010 version no. 5.0<br>EllipTrack https://github.com/tianchengzhe/EllipTrack

For manuscripts utilizing custom algorithms or software that are central to the research but not yet described in published literature, software must be made available to editors and reviewers. We strongly encourage code deposition in a community repository (e.g. GitHub). See the Nature Research guidelines for submitting code & software for further information.

Data

Policy information about availability of data

All manuscripts must include a data availability statement. This statement should provide the following information, where applicable:

- Accession codes, unique identifiers, or web links for publicly available datasets
- A list of figures that have associated raw data
- A description of any restrictions on data availability

Raw and processed scRNA-seq datasets are deposited in Gene Expression Omnibus (Accession Number: GSE164614). Source data are provided with this paper. Raw and processed data are also available upon request.

Field-specific reporting

Please select the one below that is the best fit for your research. If you are not sure, read the appropriate sections before making your selection.

☒ Life sciences ☐ Behavioural & social sciences ☐ Ecological, evolutionary & environmental sciences

For a reference copy of the document with all sections, see nature.com/documents/nr-reporting-summary-flat.pdf

Life sciences study design

All studies must disclose on these points even when the disclosure is negative.

Sample size	Sample size was not predetermined. For single cell analysis (live-cell imaging, immunofluorescence, FISH), the number of cells in each replicates were analyzed in this study. For each condition, there are usually more than 500 cells, which is sufficient to make clear conclusion. Additionally, for imaging experiments, 96-well plate formats were used, where at least 6 wells, 4 sites/well were used as technical replicates per condition tested.
Data exclusions	In scRNA-seq section, we excluded cells having proliferation probability greater or equal than exp(-40) but strictly less than 1 from the analysis since a reliable classification might not be possible.
Replication	All attempts at replication were successful. At least two independent repeats were preformed for all experiments in this paper.
Randomization	Not relevant to our study since we seed cells into a 96-well plate and image the whole plate with automated microscopy.
Blinding	N/A. The extracted features are objective.

Reporting for specific materials, systems and methods

We require information from authors about some types of materials, experimental systems and methods used in many studies. Here, indicate whether each material, system or method listed is relevant to your study. If you are not sure if a list item applies to your research, read the appropriate section before selecting a response.

Materials & experimental systems

n/a	Involved in the study	
☐	☒	Antibodies
☐	☒	Eukaryotic cell lines
☒	☐	Palaeontology and archaeology
☒	☐	Animals and other organisms
☒	☐	Human research participants
☒	☐	Clinical data
☒	☐	Dual use research of concern

Methods

n/a	Involved in the study	
☒	☐	ChIP-seq
☐	☒	Flow cytometry
☒	☐	MRI-based neuroimaging

Antibodies

Antibodies used

Phospho-Rb (Ser807/811) Cell Signaling Technology Cat#8516P
Phospho-p44/42 MAPK (ERK1/2) (Thr202/Tyr204) Cell Signaling Technology Cat#4370
ATF4 Cell Signaling Technology Cat#11815S
Phospho-Rb (Ser780) BD Biosciences Cat#558385
Phospho-S6 (S240/244) Cell Signaling Technology Cat#2215
AXL Cell Signaling Technology Cat#8661
MITF Abcam Cat#ab3201
NGFR Cell Signaling Technology Cat#8238
SOX10 Cell Signaling Technology Cat#89356

FANCD2 Novus Biologicals Cat#NB100-182
γH2AX Cell Signaling Technology Cat#9718
MCM2 (BM28) BD Biosciences Cat#610700
GAPDH(D16H11)XP Cell Signaling Technology Cat#5174
Anti-rabbit IgG HRP-linked Cell Signaling Technology Cat#7074S
Anti-rabbit Alexa Fluor-647 Thermo Fisher Cat#A-21245
Anti-rabbit Alexa Fluor-488 Thermo Fisher Cat#A-11034
Anti-mouse Alexa Fluor-488 Thermo Fisher Cat#A-11029
Anti-mouse Alexa Fluor-546 Thermo Fisher Cat#A-11030

Validation

All antibodies were validated by the manufacturers.
Phospho-Rb (Ser807/811) Antibody detects endogenous levels of Rb when phosphorylated at serine 807/811. The antibody may cross-react with Rb phosphorylated at Ser608. Species Reactivity: Human, Rat, Monkey

Phospho-p44/42 MAPK (Erk1/2) (Thr202/Tyr204) Antibody detects endogenous levels of p44 and p42 MAP Kinase (Erk1 and Erk2) when phosphorylated either individually or dually at Thr202 and Tyr204 of Erk1 (Thr185 and Tyr187 of Erk2). The antibody does not cross-react with the corresponding phosphorylated residues of either JNK/SAPK or p38 MAP Kinase, and does not cross-react with non-phosphorylated Erk1/2. Species Reactivity: Human, Mouse, Rat, Hamster, Monkey, Mink, D. melanogaster, Zebrafish, Bovine, Pig, C. elegans

ATF-4 (D4B8) Rabbit mAb recognizes endogenous levels of total ATF-4 protein. Species Reactivity:Human, Mouse, Rat

The J146-35 monoclonal antibody recognizes Rb phosphorylated at serine 780 (pS780).

Phospho-S6 Ribosomal Protein (Ser240/244) Antibody detects endogenous levels of ribosomal protein S6 only when phosphorylated at serines 240 and 244. This antibody does not detect S6 ribosomal protein phosphorylated at other sites. Species Reactivity:Human, Mouse, Rat, Monkey, Zebrafish

Axl (C89E7) Rabbit mAb detects endogenous levels of total Axl protein and does not cross-react with Tyro3.Species Reactivity:Human, Monkey

MITF: Tested applications Suitable for: ICC/IF, WB, Flow CytSpecies reactivity Reacts with: Dog, Human. Predicted to work with: Mouse

NGFR: p75NTR (D4B3) XP® Rabbit mAb recognizes endogenous levels of total p75NTR protein.Species Reactivity:Human, Mouse, Rat

Sox10 (D5V9L) Rabbit mAb recognizes endogenous levels of total Sox10 protein. This antibody also recognizes presumptive sumoylated forms of Sox10 protein. Species Reactivity:Human

This FANCD2 Antibody reacts with human and mouse FANCD2, with Primate and Canine reactivity reported in literature. Zebrafish reactivity reported in scientific literature (PMID: 30540754). Rat reactivity reported in multiple pieces of scientific literature. Kangaroo reactivity reported in scientific literature (PMID: 24982423).

Phospho-Histone H2A.X (Ser139) (20E3) Rabbit mAb detects endogenous levels of H2A.X only when phosphorylated at serine 139. Species Reactivity:Human, Mouse, Rat, Monkey

MCM2:ReactivityHuman (QC Testing) Mouse, Rat, Dog, Chicken (Tested in Development) ApplicationWestern blot (Routinely Tested) Immunofluorescence, Immunoprecipitation (Tested During Development) Immunohistochemistry (Not Recommended)

GAPDH (D16H11) XP® Rabbit mAb detects endogenous levels of total GAPDH protein. Species Reactivity:Human, Mouse, Rat, Monkey

Eukaryotic cell lines

Policy information about cell lines

Cell line source(s)

A375 was purchased from ATCC. WM278 and WM164 were obtained from Dr. Natalie Ahn at the University of Colorado Boulder. SKMEL 28, SKMEL267c, and SKMEL19 were from Dr. Neal Rosen at the Memorial Sloan Kettering Cancer Center . MB3883 and MB4562 cells were obtained from the Cutaneous Oncology Melanoma Bank at the University of Colorado.

Authentication

A375 and WM278 cells were authenticated by STR profiling as exact matches to A375 and WM278, respectively.

Mycoplasma contamination

A375 and WM278 tested negative. All other cell lines were not tested for mycoplasma.

Commonly misidentified lines
(See ICLAC register)

No commonly misidentified cell lines were used.

Flow Cytometry

Plots

Confirm that:

☒ The axis labels state the marker and fluorochrome used (e.g. CD4-FITC).

☒ The axis scales are clearly visible. Include numbers along axes only for bottom left plot of group (a 'group' is an analysis of identical markers).

☒ All plots are contour plots with outliers or pseudocolor plots.

☒ A numerical value for number of cells or percentage (with statistics) is provided.

Methodology

Sample preparation	Cells from culture were trypsinized and harvested to create cell suspensions in replicates, which were either stained live (Annexin V apoptosis assay) or processed for chromatin extraction and fixed before immunostaining (MCM2 licensing assay). Details on staining in the methods section.
Instrument	BD FACSCelesta
Software	FACSDiva (BD Biosciences, 8.0.1.1) was used to collect FCS files, which were further analyzed using FlowJo (FlowJo LLC, 10.6.1).
Cell population abundance	At the time of aquisition on FACSDiva software, cell population data were collected on a debris exclusion gate and a doublet exclusion gate, totaling at least 5,000 cell events per sample.
Gating strategy	Conventional gating strategy was employed in FlowJo to isolate reliable single-cell events for both flow cytometry assays used in the study. For both flow cytometry assays, debris-exclusion gate was determined on FSC-A vs. SSC-A. For the apoptosis assay, doublet-exclusion gate was determined on SSC-W vs. SSC-A. For MCM2 licensing assay doublet-exclusion gate was determined on Hoechst 33342 intensity (pulse area) vs. Hoechst 33342 intensity (pulse height), as shown in Supplementary Fig. 8c. Further specific gating was required for the MCM2 licensing assay, which is shown in detail in Supplementary Fig. 8d-g.

☒ Tick this box to confirm that a figure exemplifying the gating strategy is provided in the Supplementary Information.
